# Hypoglycaemia, Abnormal Lipids, and Cardiovascular Disease among Chinese with Type 2 Diabetes

**DOI:** 10.1155/2015/862896

**Published:** 2015-10-04

**Authors:** Yijun Li, Yiming Mu, Qiuhe Ji, Qin Huang, Hongyu Kuang, Linong Ji, Xilin Yang

**Affiliations:** ^1^Department of Endocrinology, Chinese PLA General Hospital, Beijing 100853, China; ^2^Department of Endocrinology, Xijing Hospital, 4th Military Medical University, Xi'an, Shaanxi 710032, China; ^3^Department of Endocrinology, Changhai Hospital of Shanghai, Shanghai 200433, China; ^4^Department of Endocrinology, The Affiliated Hospital of Harbin Medical University, Harbin, Heilongjiang 150001, China; ^5^Department of Endocrinology, Peking University People's Hospital, Beijing 100096, China; ^6^Department of Epidemiology and Biostatistics, School of Public Health, Tianjin Medical University, Tianjin 300070, China

## Abstract

We recruited a group of 6713 consecutive Chinese patients with T2D but normal renal and liver function who were admitted to one of 81 top tertiary care hospitals in China. Mild hypoglycaemia was defined as having symptomatic hypoglycaemia in one month before hospitalization. Severe hypoglycaemia was defined as having hypoglycaemia that needed assistance from other people in three months before hospitalization. Prior cardiovascular disease (CVD) was defined as having coronary heart disease, stroke, or peripheral arterial disease. Of 6713 patients, 80 and 304 had severe and mild hypoglycaemia episodes, respectively, and 561 had CVD. Patients with severe and mild hypoglycaemia episodes were more likely to have prior CVD (32.5% versus 16.5% versus 7.7%, *P* < 0.0001). Both mild and severe hypoglycaemia were associated with increased risk of CVD (adjusted odds ratios (ORs): 2.64, 95% CI: 1.85–3.76 for mild hypoglycaemia; 6.59, 95% CI: 3.79–11.45 for sever hypoglycaemia) than those patients free of hypoglycaemia. Further adjustment for lipid profile did not change these two ORs. In the same way, the ORs of lipid profile for CVD were similar before and after adjustment for hypoglycaemia. We concluded that hypoglycaemia and lipid profile were independently associated with increased risk of CVD.

## 1. Introduction

Hypoglycaemia is the most common acute episode in the management of type 2 diabetes (T2D) [1]. Recent several large randomised clinical trials (RCT) rekindled a strong interest in hypoglycaemia. In the ACCORD (Action to Control Cardiovascular Risk in Diabetes) [2], the intensive management aiming at achieving glycated haemoglobin (HbA1c) target (<6.0%) resulted in more frequent hypoglycaemia that needed assistance and unexpected more deaths in the intensive management arm. The ADVANCE (Action in Diabetes and Vascular Disease: PreterAx and DiamicroN Modified Release Controlled Evaluation) also found that intensive glucose control aiming at achieving HbA1c <6.5% led to more frequent severe hypoglycaemia (2.7% versus 1.5% in the standard-control group; hazard ratio, 1.86; 95% CI, 1.42 to 2.40) but all-cause mortality was similar in both groups [3]. The Veterans Affairs Diabetes Trial (VADT) [4] also reported significantly more episodes in the intensive-therapy group than in the standard-therapy group (*P* < 0.001). The epidemiological analysis of the ACCORD trial data [5] found that symptomatic severe hypoglycaemia was associated with an increased risk of death in both the intensive glucose control arm and the standard glucose control arm. Similarly, epidemiological analysis of the ADVANCE trial data [6] also reported that severe hypoglycaemia was associated with a significant increase in the risks of macrovascular and microvascular events and death from cardiovascular and any causes. These findings from epidemiological analysis of trial data are consistent from observational studies [7, 8]. Additionally, hypoglycaemic episodes may increase the risk of cardiac arrhythmias [9], dementia [10], and accidents [11]. However, detailed analysis of the ACCORD trial data suggested that the increased death risk in the intensive glucose arm could not be attributable to more frequent hypoglycaemia [5], and the authors of the ADVANCE group [6] also suggested that hypoglycaemia might be a marker of vulnerability to those clinical events. Indeed, the data of Hong Kong Diabetes Registry [12] suggested that severe hypoglycaemia that needed hospitalization identified vulnerable patients with T2D at high risk of cancer and mortality due to sharing some cancer phenotypes including low low-density lipoprotein cholesterol (LDL-C) plus low triglyceride [13] and low high-density lipoprotein cholesterol (HDL-C) [14]. Also noted is that a recent meta-analysis of cohort studies suggests that the association between hypoglycaemia and cardiovascular disease could not be entirely attributable to comorbid severe illness [8]. It remains to be known whether hypoglycaemia and abnormal lipid profile are independent in their associations with cardiovascular disease (CVD). Thus, our study aimed to investigate whether symptomatic or severe hypoglycaemia and abnormal lipid profile identify different groups at increased risk of CVD, that is, both having independent associations with CVD.

## 2. Methods

### 2.1. Patients

In 2013, Chinese Hospital Association (CHA) set up a systematic management program of hyperglycemia in inpatients with type 2 diabetes (T2D) admitted to top tertiary hospital to improve the care of inpatients with T2D in China and, in particular, to learn the profile of hypoglycaemia and associated factors. A total of 81 top tertiary care hospitals in 27 cities from 21 provinces were invited and agreed to participate in the study. The inclusion criteria were as follows: (1) patients with T2D admitted to the department of endocrinology; (2) using a basal bolus plus meal time insulin insensitive management scheme; and (3) being between 18 and 80 years of age. The exclusion criteria were as follows: (1) liver dysfunction defined as alanine aminotransferase (ALT) or aspartate aminotransferase (AST) ≥2.5-fold of the upper limits of the normal range, 0–40 U/L; (2) renal dysfunction defined as serum creatinine ≥125 *μ*mol/L in male and ≥110 *μ*mol/L in female or chronic kidney disease (CKD); (3) pregnancy or lactation; and (4) inability to communicate in a normal way.

From May 2013 to August 2013, we successfully recruited 6713 patients with T2D from the 81 hospitals and used them in the final analysis. Ethical approval was obtained from the People's General Army (PLA) Hospital Clinical Research Ethics Committee and written informed consent was obtained from all patients for data analysis and research purpose.

### 2.2. Clinical Measurements

Measured parameters included body weight, body height, and sitting BP (after 5 minutes of rest). Fasting blood was taken for measurement of glycated hemoglobin (HbA_1c_), lipids including total cholesterol, low-density lipoprotein cholesterol (LDL-C), high-density lipoprotein cholesterol (HDL-C), triglyceride, renal and liver function, complete blood count, and HbA_1c_. The first-morning urine was used to calculate urinary albumin to creatinine ratio (ACR). Albuminuria was defined as ACR ≥2.5 mg/mmol in men and ≥3.5 mg/mmol in women. The abbreviated Modification of Diet in Renal Disease (MDRD) Study formula recalibrated for Chinese [15] was used to estimate glomerular filtration rate by the following equation: estimated GFR = 186 × [SCR × 0.011]^−1.154^ × [age]^−0.203^ × [0.742  if  female] × 1.233, where SCR is serum creatinine expressed as *μ*mol/L (original mg/dL converted to *μ*mol/L) and 1.233 is the adjusting coefficient for Chinese [16]. Chronic kidney disease (CKD) was defined as eGFR <60 mL/min/1.73 m^2^. The cut-off points recommended by the American Diabetes Association (ADA) [17] were used to define abnormal lipids, that is, LDL cholesterol <2.6 mmol/L, triglyceride <1.7, and HDL cholesterol ≥1.0 mmol/L in male and ≥1.3 mmol/L in female.

### 2.3. Definition of Hypoglycaemia and Cardiovascular Disease

Patients were asked whether they had asymptomatic hypoglycaemia defined as plasma glucose ≤3.9 mmol/L but without any symptoms in one month before hospitalization, whether they had symptomatic hypoglycaemia with or without plasma glucose ≤3.9 mmol/L in one month before hospitalization, and whether they had severe hypoglycaemia defined as having hypoglycaemia that needed assistance from other people in three months before hospitalization. In this study, mild hypoglycaemia was defined as symptomatic hypoglycaemia in one month before hospitalization and severe hypoglycaemia defined as hypoglycaemia that needed assistance from other people in three months before hospitalization. A prior coronary heart disease (CHD) was defined as having CHD. CHD included myocardial infarction, ischemic heart disease, coronary revascularization, percutaneous transluminal coronary angioplasty, or coronary atherectomy. Similarly, a prior stroke (but not transient ischemic attack) before or at this visit was defined as having stroke no matter whether the stroke was completely or incompletely recovered. Peripheral arterial disease (PAD) was defined by lower limb amputation, revascularization for PAD, or absence of foot pulses as confirmed by an ankle : brachial ratio <0.90 measured by Doppler ultrasound examination. In this study, CVD was defined as having either CHD or stroke or PAD.

### 2.4. Statistical Analysis

The Statistical Analysis System (Release 9.30; SAS Institute, Cary, NC) was used to analyse the data. All continuous variables were expressed as mean (standard error, SE) or median (interquartile range, IQR) as appropriate while categorical variables were expressed as percentages (number, *n*). Body mass index (BMI) was calculated as body weight in kilograms divided by squared body height in meters. Normality of distribution of continuous variables was checked using Q-Q plots. Log transformation of continuous variables was performed before comparisons if they were not normally distributed.

General linear model was used to perform analysis of variance to compare the differences among the three groups of patients with mild hypoglycaemia and severe hypoglycaemia and without mild or severe hypoglycaemia. Bonferroni test was used to adjust for multiple comparisons. The binary logistic regression was used to obtain odds ratios (ORs) of severe hypoglycaemia and mild hypoglycaemia versus nonhyperglycemia for CVD before and after adjusting for levels of LDL-C, HDL-C, and triglyceride as well as ORs of LDL-C, HDL-C, and triglyceride for CVD before and after adjusting for hypoglycaemia. This procedure was also used to obtain ORs of abnormal lipids for either severe hypoglycaemia or mild hypoglycaemia. The generalized logit model was used to obtain ORs of abnormal lipids for severe hypoglycaemia and mild hypoglycaemia, respectively. A structured adjustment scheme was used to adjust for confounding effects of other variables, as shown in Tables 2 and 3.

As adjustment for use of lipid lowering drugs might fail to completely remove the confounding effects of use of these drugs, we further perform a sensitivity analysis for the main analysis after exclusion of 408 users of lipid lowering drugs.

## 3. Results 

### 3.1. Characteristics of the Study Patients

Patients with severe hypoglycaemia had an older age but those with mild hypoglycaemia had a younger age than those without hypoglycaemia. Patients with severe hypoglycaemia or mild hypoglycaemia had a longer duration of disease and lower BMI than patients without. The percentages of obesity and overweight were decreased steadily from nonhypoglycaemia to mild hypoglycaemia and then to hypoglycaemia. HbA1c was observed to be lower in mild hypoglycaemia than in severe hypoglycaemia. There were marked increases in the levels of LDL-C and triglyceride and the percentages of high LDL-C and high triglyceride from nonhypoglycaemia to mild hypoglycaemia and then to hypoglycaemia. However, the level of HDL-C was also increased and the percentage of low HDL-C decreased from nonhypoglycaemia to mild hypoglycaemia and then to hypoglycaemia. A total of 561 patients had prior CVD, that is, CHD, stroke, and PAD. There were marked increases in the percentages of CVD from nonhypoglycaemia to mild hypoglycaemia and then to hypoglycaemia (Table 1).

### 3.2. Abnormal Lipids for Hypoglycaemia

Increased LDL-C was associated with mild hypoglycaemia, severe hypoglycaemia, or both in multivariable analysis. The OR of LDL-C per mmol/L was 1.17 (95% CI: 1.04–1.31) in multivariable analysis. However, increased but not decreased HDL-C was associated with increased risk of mild hypoglycaemia (OR per mmol/L: 1.10, 95% CI: 1.03–1.17) and either mild or severe hypoglycaemia (OR: 1.09, 95% CI: 1.02–1.16) but not associated with severe hypoglycaemia (Table 2).

### 3.3. Hypoglycaemia for Cardiovascular Disease

Severe hypoglycaemia and, to a lesser extent, mild hypoglycaemia were associated with increased risks of CVD in univariable and multivariable analyses. After adjusting for traditional risk factors and drug use, patients with severe hypoglycaemia were more likely to have developed CVD (OR: 6.59, 95% CI: 3.79–11.45) than those patients free of hypoglycaemia. Patients with mild hypoglycaemia were also at markedly increased risk of CVD than those patients without hypoglycaemia (OR: 2.64, 95% CI: 1.85–3.76). Further adjustment for lipids did not change the ORs of hypoglycaemia for CVD (Table 3).

### 3.4. Lipids for Cardiovascular Disease

Increased LDL-C and triglyceride and decreased HDL-C were associated with increased risk of CVD in univariable analysis. After adjusting for traditional risk factors and drug use, decreased HDL-C and increased triglyceride were still significantly associated with CVD (OR of HDL-C per mmol/L: 0.89, 95% CI: 0.80–0.99; OR of triglyceride per mmol/L: 1.08, 95% CI: 1.02–1.14). Further adjusting for hypoglycaemia only slightly changed the sizes of ORs of HDL-C and triglyceride (Table 3).

### 3.5. Sensitivity Analysis

In the sensitivity analysis, exclusion of lipid lowering drug users slightly enhanced the associations between hypoglycaemia and CVD while it did not change the effect sizes of lipids for CVD although the latter did not reach statistical significance (Table 4).

## 4. Discussions

Our study found that although levels of lipids were associated with hypoglycaemia among Chinese patients with T2DM, hypoglycaemia and levels of lipids were independently associated with increased risk of CVD, that is, the association between hypoglycaemia and CVD not being attenuated by levels of lipids and the association between levels of lipids and CVD not being attenuated by hypoglycaemia.

Many studies [7, 8] including the epidemiological analysis of the large trial data [5, 6] reported that severe hypoglycaemia was associated with increased risk of cardiovascular disease and mortality. Consistently, our findings confirmed that Chinese T2D patients with normal kidney dysfunction and liver function who had either mild or severe hypoglycaemia were associated with markedly increased risk of cardiovascular disease. On the other hand, there were a few studies that investigated risk factors for hypoglycaemia or demographic and clinical features of patients with hypoglycaemia. For example, advanced age, low BMI, poor glycaemic control [12], cognitive impairment, current use of sulphonylureas and current insulin use [18], and renal failure [12, 18, 19] were reported to be associated with hypoglycaemia. Even few studies investigated whether hypoglycaemia is characterized by abnormal lipids. In this regard, a Hong Kong group reported that cancer subphenotypes including low LDL-C, low triglyceride, or low HDL-C as well as copresence of low LDL-C and low triglyceride predicted hospital admission due to hypoglycaemia among Hong Kong Chinese with T2D [12]. In our study, increased LDL-C was associated with increased risk of CVD. However, unexpectedly, increased HDL-C but not decreased HDL-C was associated with increased risk of CVD. Of note, the data from Hong Kong showed that severe hypoglycaemia was associated with increased risk of cancer specific death but not associated with CVD specific death [20]. Thus, the data of Hong Kong study were likely to suggest that shared lipid profile of hypoglycaemia and cancer may be responsible for the increased risk of hypoglycaemia and cancer [12]. In this regard, our data support the findings of the meta-analysis [8] that hypoglycaemia was a risk factor for CVD, independently of traditional CVD risk factors and comorbid severe illness. The major differences between the Hong Kong Diabetes Registry and the current study were as follows: (1) the Hong Kong Diabetes Registry is long term cohort study while our study is a cross-sectional study, and reverse associations between hypoglycaemia and abnormal lipids could be excluded; (2) CKD, a strong risk factor for hypoglycaemia, accounted for 10% of the patients but in our study these high risk patients were excluded but patients with cancer were excluded in our current survey; (3) the severe hypoglycaemia was defined by hospitalization due to hypoglycaemia but only 76 out of 384 patients reporting to have had mild or severe hypoglycaemia were hospitalised due to hypoglycaemia. These differences may partially account for the different findings in Hong Kong Diabetes Registry and our survey.

Typical abnormal lipids in T2D are increased levels of triglyceride, decreased levels of HDL-C, and increased levels of small dense LDL particles, due to increased free fatty acid flux subsequent to insulin resistance [21, 22]. Hyperglycemia led to albuminuria that was strongly predictive of end-stage renal disease [23, 24]. On the other hand, albuminuria increased the risk of high LDL-C but chronic kidney disease (CKD) increased the risk of low HDL-C [25] while albuminuria and CKD also modified the associations of total cholesterol (or LDL-C) and HDL-C with CHD [26]. Our study observed associations between LDL-C/HDL-C and hypoglycaemia but adjustment for hypoglycaemia did not attenuate the associations between lipids and CVD, suggesting that hypoglycaemia could not explain the increased risk of CVD with abnormal lipid profile, that is, not suggesting that abnormal lipids and hypoglycaemia had causal relationships between each other. In addition, we also noticed that patients with hypoglycaemia were an undertreated group, for example, few with severe hypoglycaemia taking statins, which might contribute to the observed associations between lipids and hypoglycaemia.

Hypoglycaemia is one of the most common acute complications that plays an important role in achieving optimal glycaemic control [27]. Although some drug treatments such as use of insulin and secretagogues [28] are associated with higher rates of hypoglycaemia, occurrence of hypoglycaemia itself may contribute to increased risk of CVD as well as multidimensional impairment [29] in T2D. Given the increasing prevalence of diabetes in China [30], it is critical to reduce the rates of diabetes complications in these high-risk patients to reduce the burden of disease. Clinicians need to balance the benefits of tight glycaemic control [31, 32] and possible harms of hypoglycaemia associated with tight glycemia control [2–4] in the management of T2D.

Our study has several limitations. First, this study was a cross-sectional survey of inpatients being hospitalised due to T2D. The study cannot establish time relationship regarding the associations between hypoglycaemia and abnormal lipids. Second, patients with abnormal liver function or CKD were excluded. Albuminuria was the strongest predictor of renal endpoint [33] and most of the patients with albuminuria and high BP might have been excluded due to exclusion of patients with CKD. Thus, our study could not examine the associations of urinary ACR, eGFR, or BP with hypoglycaemia, which had been shown to be associated with hypoglycaemia in Hong Kong Diabetes Registry [12]. Third, smoking and alcohol drinking habits were not collected in this survey. Their confounding effects cannot be adjusted. Fourth, the patients were patients being hospitalised due to T2D and the findings of this survey need to be confirmed in low risk patients with T2D. Fifth, it is noticed that there was much greater intraindividual variability in hypoglycaemia symptom reporting by patients with diabetes over a long period, that is, 12-month period [34]. Although we chose reporting hypoglycaemia symptoms over more recent short periods of time, intraindividual variability in reporting these symptoms by our subjects was unavoidable.

In conclusion, in a large survey of Chinese inpatients with T2D, we found that hypoglycaemia and lipid profile were independent risk factors for CVD and both factors may be useful in assessing CVD risk among Chinese patients with T2D. Further studies are warranted to validate these findings in cohorts of patients with T2D, including low risk Chinese with T2D.

## Figures and Tables

**Table 1:** Clinical and biochemical characteristics of study patients by hypoglycaemia.

Variables	Nonhypoglycaemia (*n* = 6329)	Mild hypoglycaemia (*n* = 304)	Severe hypoglycaemia (*n* = 80)	*P* value
	Mean or % (S.E. or n)	Mean or % (S.E. or *n*)	Mean or % (S.E. or *n*)	
Age, year	56.4 (0.13)^†,‡^	55.5 (0.60)^†^	61.5 (1.18)^‡^	<0.0001
Male gender	56.7% (3589)	52.0% (158)	62.5% (50)	0.1490
BMI, kg/m^2^	24.0 (0.03)^†,‡^	22.7(0.18)^†,§^	21.7(0.35)^‡,§^	<0.0001
BMI ≥ 24.0 but <28 kg/m^2^	39.5% (2497)	22.7% (69)	10.0% (8)	<0.0001
BMI ≥ 28 kg/m^2^	9.2% (580)	7.2% (22)	0% (0)	
Duration of diabetes, year^*^	3.6 (0.05)^†^	5.0 (0.25)^†^	4.4 (0.48)	<0.0001
Systolic BP, mmHg	131 (0.2)	131 (0.7)	131 (1.3)	0.8935
Diastolic BP, mmHg	81 (0.1)	81 (0.4)	81 (0.9)	0.6782
HbA1c, %	10.4 (0.02)^†^	10.0 (0.09)^†^	10.3 (0.17)	0.0006
LDL-C, mmol/L	3.15 (0.01)^†,‡^	3.36 (0.06)^†^	3.64 (0.11)^‡^	0.0001
LDL-C ≥ 2.6 mmol/L	80.0% (5064)	89.1% (271)	96.3 (77)	<0.0001
HDL-C, mmol/L^*^	1.90 (1.39–2.90)^†,‡^	2.60 (1.90–3.20)^†^	2.50 (2.00–2.90)^‡^	<0.0001
HDL-C <1.0 mmol/L in male and <1.3 mmol/L in female	14.6% (924)	6.9% (21)	0% (0)	<0.0001
Triglyceride, mmol/L^*^	2.30 (1.54–3.00)^†,‡^	2.60 (2.15–3.20)^†^	2.80 (2.40–3.20)^‡^	<0.0001
Triglyceride ≥ 1.7 mmol/L	70.8% (4483)	85.2% (259)	97.5% (78)	<0.0001
Urinary ACR, mg/mmol^*^	0.17 (0.15–0.20)^†^	0.16 (0.15–0.19)	0.16 (0.15–0.17)^†^	0.0025
Complications				
Coronary artery disease	4.9% (308)	11.8% (36)	16.3% (13)	<0.0001
Stroke	1.3% (85)	2.6% (8)	16.3% (13)	<0.0001
Peripheral artery disease	3.1% (199)	3.6% (11)	7.5% (6)	0.0830
Cardiovascular diseases	7.7% (485)	16.5% (50)	32.5% (26)	<0.0001
Drug use before admission				
Statins	5.2% (327)	5.3% (16)	1.3% (1)	0.2857
Other lipid lowering drugs	0.9% (59)	0.7% (2)	3.8% (3)	0.0312
Renin-angiotensin system inhibitors	5.9% (370)	8.2% (25)	3.8% (3)	0.1629
Other antihypertensive drugs	3.0% (190)	4.9% (15)	0% (0)	0.0449
Oral antidiabetes drugs only	38.9% (2461)	49.0% (149)	38.8% (31)	0.0019
GLP-1 based treatment	0.2% (13)	0.7% (2)	15% (12)	<0.0001
Basal insulin based treatment				<0.0001
No	87.3% (5524)	77.3% (235)	80.0% (64)	
Basal insulin	7.4% (471)	17.4% (53)	14% (17.5%)	
Basal + meal time insulin	5.3% (334)	5.3% (16)	2.5% (2)	
Premixed insulin based treatment				<0.0001
No	79.3% (5016)	81.6% (248)	73.8% (59)	
Once per day	0.8% (48)	1.0% (3)	16.3% (13)	
Twice per day	20.0% (1265)	17.4% (53)	10.0% (8)	

ACR: albumin to creatinine ratio; GLP: glucagon-like peptide; BMI: body mass index; BP: blood pressure; LDL-C: low-density lipoprotein cholesterol; HDL-C: high-density lipoprotein cholesterol.

^*^Data were presented as median and their interquartile ranges.

*P* values were derived from Chi-square test or analysis of variance. For analysis of continuous variables, Bonferroni test was used to perform multiple comparisons with identical †, ‡, or § indicating statistically significant differences between two means.

**Table 2:** Odds ratio of abnormal lipids for mild and severe hypoglycaemia.

	Mild hypoglycaemia		Severe hypoglycaemia		Either mild or severe hypoglycaemia	
	OR (95% CI)	*P* value	OR (95% CI)	*P* value	OR (95% CI)	*P* value
Model one						
LDL-C, mmol/L	1.12 (1.00–1.26)	0.0526	1.22 (1.05–1.43)	0.0139	1.14 (1.03–1.26)	0.0109
HDL-C, mmol/L	1.12 (1.00–1.26)	0.0526	1.22 (1.05–1.43)	0.0139	1.14 (1.03–1.26)	0.0109
Triglyceride, mmol/L	1.12 (1.05–1.19)	0.0002	1.02 (0.87–1.19)	0.7988	1.10 (1.05–1.17)	0.0005
Model two						
LDL-C, mmol/L	1.14 (1.01–1.29)	0.0331	1.21 (1.04–1.17)	0.0163	1.15 (1.03–1.28)	0.0110
HDL-C, mmol/L	1.10 (1.04–1.17)	0.0015	1.01 (0.87–1.17)	0.8922	1.09 (1.03–1.16)	0.0049
Triglyceride, mmol/L	0.96 (0.86–1.07)	0.4664	1.10 (0.94–1.28)	0.2313	0.98 (0.89–1.09)	0.7388
Model three						
LDL-C, mmol/L	1.14 (1.01–1.29)	0.0340	1.24 (1.05–1.48)	0.0129	1.17 (1.04–1.31)	0.0079
HDL-C, mmol/L	1.10 (1.03–1.17)	0.0030	1.02 (0.83–1.24)	0.8860	1.09 (1.02–1.16)	0.0084
Triglyceride, mmol/L	0.92 (0.82–1.04)	0.1858	1.02 (0.82–1.26)	0.8916	0.93 (0.83–1.04)	0.2238

Model one: not adjusted for other variables.

Model two: adjusted for age, sex, BMI, HbA1c, systolic BP, and log-transformed urinary albumin to creatinine ratio.

Model three: further adjusted for diabetes complications (coronary artery disease, stroke, and peripheral arterial disease) and drug use (statins, other lipid lowering drugs, rennin-angiotensin system inhibitors, oral antidiabetes drugs only, glucagon-like peptide-1 based treatment, basal insulin based treatment, and premixed insulin based treatment).

**Table 3:** Odds ratio of hypoglycaemia and lipid profiles for cardiovascular disease.

	OR (95% CI)	*P* value

Hypoglycaemia for CVD		
Model one		<0.0001
Nonhyperglycemia	Reference	
Mild hyperglycemia	2.37 (1.72–3.26)	
Severe hyperglycemia	5.80 (3.60–9.35)	
Model two		<0.0001
Nonhyperglycemia	Reference	
Mild hyperglycemia	2.64 (1.85–3.76)	
Severe hyperglycemia	6.59 (3.79–11.45)	
Model three		
Nonhyperglycemia	Reference	<0.0001
Mild hyperglycemia	2.64 (1.85–3.76)	
Severe hyperglycemia	6.59 (3.79–11.45)	
Lipid profile for CVD		
Model four		
LDL cholesterol, mmol/L	1.11 (1.01–1.24)	0.0351
HDL cholesterol, mmol/L	0.69 (0.63–0.77)	<0.0001
Triglyceride, mmol/L	1.12 (1.06–1.78)	<0.0001
Model five		
LDL cholesterol, mmol/L	0.95 (0.84–1.07)	0.3703
HDL cholesterol, mmol/L	0.89 (0.80–0.99)	0.0299
Triglyceride, mmol/L	1.08 (1.02–1.14)	0.0160
Model six		
LDL cholesterol, mmol/L	0.93 (0.82–1.05)	0.2462
HDL cholesterol, mmol/L	0.87 (0.78–0.97)	0.0097
Triglyceride, mmol/L	1.08 (1.01–1.14)	0.0166

Model one: not adjusted for other variables.

Model two: adjusted for age, sex, BMI, systolic blood pressure and log-transformed urinary albumin to creatinine ratio, and drug use (statins, other lipid lowering drugs, renin-angiotensin system inhibitors, oral antidiabetes drugs [OADs] only, glucagon-like peptide-1 based treatment, basal insulin based treatment, and premixed insulin based treatment).

Model three: further adjusted for LDL-C, HDL-C, and triglyceride.

Model four: not adjusted for other variables.

Model five: adjusted for age, sex, BMI, systolic blood pressure, LDL-C, HDL-C, triglyceride and log-transformed urinary albumin to creatinine ratio, and drug use (statins, other lipid lowering drugs, renin-angiotensin system inhibitors, and oral antidiabetes drugs [OADs] only).

Model six: adjusted for the variables listed in model five and hypoglycaemia.

**Table 4:** Sensitivity analysis of odds ratio of hypoglycaemia and lipid profiles for cardiovascular disease after exclusion of 408 patients who used lipid lowering drugs.

	OR (95% CI)	*P* value

Hypoglycaemia for CVD		
Model one		<0.0001
Nonhyperglycemia	Reference	
Mild hyperglycemia	2.86 (2.04–4.02)	
Severe hyperglycemia	7.72 (4.73–12.60)	
Model two		<0.0001
Nonhyperglycemia	Reference	
Mild hyperglycemia	2.95 (2.02–4.29)	
Severe hyperglycemia	6.75 (3.79–12.04)	
Model three		<0.0001
Nonhyperglycemia	Reference	
Mild hyperglycemia	2.95 (2.02–4.29)	
Severe hyperglycemia	6.75 (3.79–12.04)	
Lipid profile for CVD		
Model four		
LDL cholesterol, mmol/L	1.06 (0.94–1.20)	0.3604
HDL cholesterol, mmol/L	0.79 (0.70–0.89)	<0.0001
Triglyceride, mmol/L	1.09 (1.02–1.16)	0.0165
Model five		
LDL cholesterol, mmol/L	0.93 (0.80–1.09)	0.3755
HDL cholesterol, mmol/L	0.91 (0.82–1.03)	0.1201
Triglyceride, mmol/L	1.07 (0.99–1.16)	0.0739
Model six		
LDL cholesterol, mmol/L	0.91 (0.77–1.07)	0.2469
HDL cholesterol, mmol/L	0.88 (0.77–1.00)	0.0580
Triglyceride, mmol/L	1.08 (1.00–1.17)	0.0593

Model one: not adjusted for other variables.

Model two: adjusted for age, sex, BMI, systolic blood pressure and log-transformed urinary albumin to creatinine ratio, and drug use (statins, other lipid lowering drugs, renin-angiotensin system inhibitors, oral antidiabetes drugs [OADs] only, glucagon-like peptide-1 based treatment, basal insulin based treatment, and premixed insulin based treatment).

Model three: further adjusted for LDL-C, HDL-C, and triglyceride.

Model four: not adjusted for other variables.

Model five: adjusted for age, sex, BMI, systolic blood pressure, LDL-C, HDL-C, triglyceride and log-transformed urinary albumin to creatinine ratio, and drug use (statins, other lipid lowering drugs, renin-angiotensin system inhibitors, oral antidiabetes drugs [OADs] only, glucagon-like peptide-1 based treatment, basal insulin based treatment, and premixed insulin based treatment).

Model six: adjusted for the variables listed in model five and hypoglycaemia.
